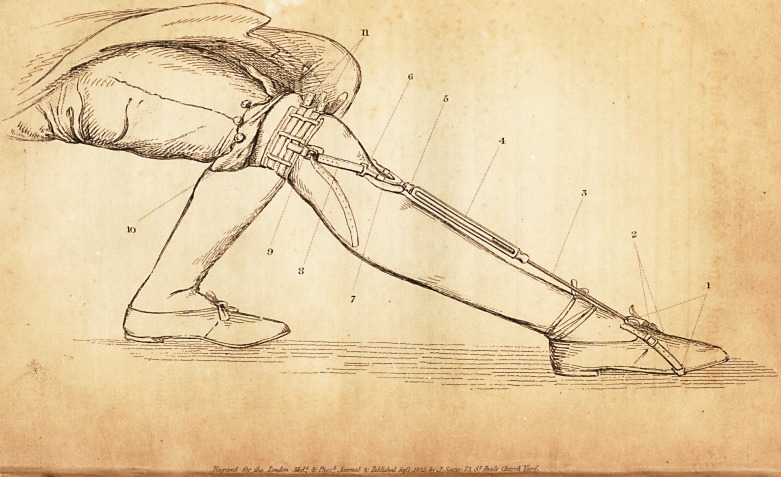# An Engraving of a New Apparatus for the Prevention and Cure of Pointed Toe

**Published:** 1823-09

**Authors:** Joseph Amesbury

**Affiliations:** Member of the Royal College of Surgeons in London, &c.


THE LONDON

### Medical and Physical Journal.

3 OF VOL. L.] SEPTEMBER, 1823. [No. 295.

For many fortunate discoveries in medicine, and for the detection of numerous errors, the world is indebted to the rapid circulation of Monthly Journals; and there never existed any work, to which the Faculty, in Europe and America, were under deeper obligations, than to the Medical and Physical Journal of London, now forming a long, but an invaluable, series.—RUSH.

### ORIGINAL COMMUNICATIONS,

### SELECT OBSERVATIONS, &c.

ART. I.—*An Engraving of a new Apparatus for the Prevention and Cure of Pointed Toe.* Invented by JOSEPH AMESBURY, Esq. Member of the Royal College of Surgeons in London, &c.

I DO myself the pleasure to forward to you an engraving of an apparatus, which I have contrived for the prevention and cure of that deformity which is usually denominated *pointed toe.* In this deformity the foot is extended upon the leg, and becomes fixed in that position. It sometimes exists at birth, but generally arises from disease or accident. The action of the apparatus is to keep the foot flexed, or to bend it when extended.

By the judicious management of this little apparatus during the cure of those diseases which produce a permanent extended state of the foot, the occurrence of the deformity may be prevented. If the toe already points, the foot may be bent upon the leg in the most gradual manner, by turning the female screws, 4. If the case be of such a nature that the patient may be allowed to be about with impunity, the apparatus in no way impedes progression. This must be regarded as an advantage in the treatment, because we are enabled to remove the affection without confining the patient from his usual avocations.

Since I had this apparatus constructed, which is now nearly two years, I have employed it in a variety of cases, in some of which it was used to prevent, and in others to cure, the deformity in question; and, as my experience of its utility furnishes me with the happiest results, I am induced to send an engraving of it for your inspection, thinking you may consider it of sufficient importance to give it publicity through the medium of your widely-circulated Journal.

The drawing was taken from a boy, fourteen years of age, who had the apparatus applied to his limb for the cure of a bad case of pointed toe. The boy was placed in the sitting posture, so as to bring the sole of the foot flat upon the ground,

The plate shows accurately the degree of deformity arising from the position of the foot, and also the manner in which the apparatus was applied.

1. Stirrup of the apparatus fixed to the sole of the shoe.
2. Straps for lengthening stirrup at pleasure.
3. Male screw fixed to the stirrup.
4. Female screw.
5. Pivot upon which the female screw turns.
6. A guard to keep off uneasy pressure from the knee.
7. Pad to the guard.
8. Straps for lengthening the apparatus, so that it may be suited to different lengths of limb.
9. Sliding buckle, padded, and retained in its proper situation by the inferior circular strap, (10.)
10. Two straps passed round the limb upon sliding pads, (11.)
11. Sliding pads placed just above the condyles of the femur, so as to prevent inconvenience from pressure upon the soft parts, and at the same time to keep the circular straps off the ham.

The surgeon, having properly adjusted the apparatus, regulates the action of the screw according to the nature of the case.

82, *Great Surrey-street.*

## Figures and Tables

**Figure f1:**